# Recombinant Human Neuregulin1‐β1 Significantly Reduces Schwannoma Growth in Mice

**DOI:** 10.1002/ana.78050

**Published:** 2025-10-17

**Authors:** Julia P. Bischoff, Alexander Schulz, Christian Hagel, Robert Büttner, Michael Reuter, Waylan K. Bessler, D. Wade Clapp, Helen Morrison

**Affiliations:** ^1^ Leibniz Institute on Aging Fritz Lipmann Institute Jena Germany; ^2^ Institute of Neuropathology University Medical Centre Hamburg‐Eppendorf Hamburg Germany; ^3^ Department of Pediatrics, Herman B Wells Center for Pediatric Research Department of Biochemistry Indiana University School of Medicine Indianapolis IN USA; ^4^ Faculty of Biological Sciences Friedrich‐Schiller University Jena Germany

## Abstract

**Objective:**

Schwannomas are benign tumors that arise from Schwann cells of the nerve sheath, and their management presents a significant clinical challenge, particularly in genetic conditions like NF2‐related schwannomatosis (NF2‐SWN). Although current treatments, including surgery, radiation, and repurposed pharmacological agents, can be effective, they are often limited by issues such as tumor recurrence and the risk of nerve function impairment. This study aims to evaluate the potential of recombinant human Neuregulin1 beta 1 (rhNRGβ1) to inhibit schwannoma growth and promote Schwann cell differentiation in preclinical models.

**Methods:**

We investigated the therapeutic potential of rhNRGβ1, a recombinant human epidermal growth factor (EGF)‐like domain of Neuregulin1 beta 1, as a growth‐inhibitory agent for schwannomas. Two distinct mouse models were used to assess its efficacy, with both histological and functional endpoints analyzed.

**Results:**

Both systemic and local administration of rhNRGβ1 resulted in a significant reduction in schwannoma tumor growth. Mechanistically, rhNRGβ1 not only inhibited tumor proliferation, but also promoted the differentiation of both proliferative and de‐differentiated Schwann cells, suggesting a dual action of growth inhibition and cellular maturation.

**Interpretation:**

These findings highlight the therapeutic potential of rhNRG1‐β1 in managing schwannomas, not only by reducing tumor growth, but also by promoting the maturation and functional restoration of Schwann cells. This dual effect provides a promising avenue for novel therapeutic strategies aimed at addressing both the growth and cellular differentiation challenges associated with schwannomas in NF2‐SWN and other related conditions. ANN NEUROL 2026;99:369–381

## Graphical abstract

rhNRGβ1‐Replacement‐Therapy: Under physiological conditions, NRGβ1 is expressed on axons (in orange), where it activates ERBB2 receptors, facilitating successful nerve regeneration following injury. However, loss of NF2 leads to a reduction in NRGβ1‐expression and increased ErbB2 levels on Schwann cells (in green), which contributes to schwannoma formation, particularly in conditions like NF2‐related schwannomatosis. The replacement of NRGβ1‐signaling using recombinant human Neuregulin1‐β1 (rhNRGβ1) represents a new strategy for schwannoma treatment. This approach reduces schwannoma growth by promoting Schwann cell differentiation and maturation.

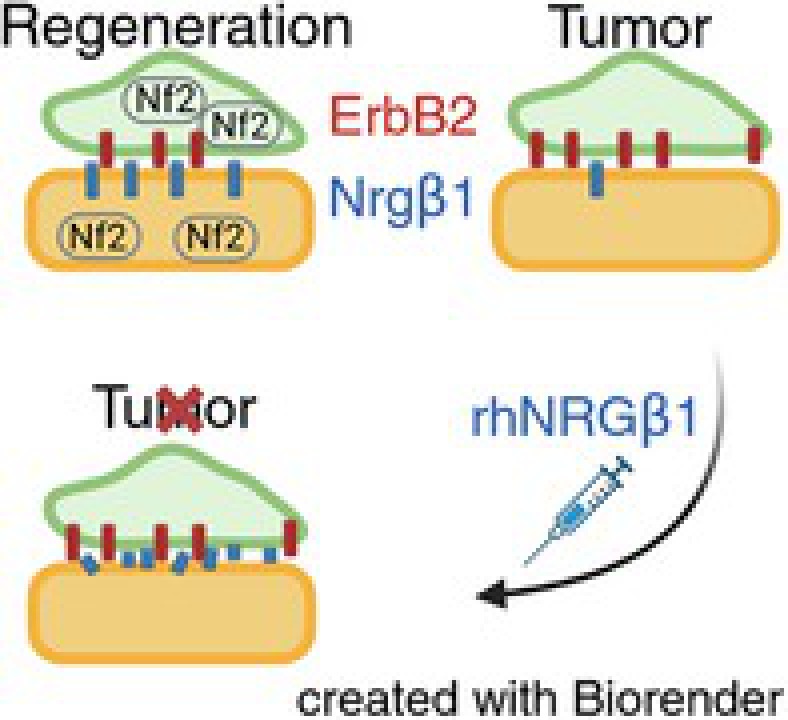

Schwannomas are Schwann cell‐derived nerve sheath tumors that arise sporadically and in association with genetic tumor predisposition syndromes, such as NF2‐related schwannomatosis (NF2‐SWN). Although non‐invasive, these tumors may impact significantly on quality of life. For instance, NF2‐SWN—a hereditary tumor syndrome with an incidence of 1 in 33,000 live births^1^—may result in lifelong deafness and carries the risk of brainstem compression, as schwannomas predominantly affect the vestibulocochlear nerve (vestibular schwannoma). The possible occurrence of multiple schwannomas in individuals with NF2‐SWN underscores the imperative for pharmacological treatments that provide long‐lasting, systemic control of such tumors.

Previous research established that the genetic basis for schwannoma formation is bi‐allelic inactivation of the NF2 gene in Schwann cells, resulting in unrestricted cell proliferation and over‐expression of growth‐promoting molecules—such as the receptor tyrosine kinase ERBB2.^2^ Earlier work from our group revealed that ErbB2 over‐expression by Schwann cells also results from NF2 gene reduction in adjacent axons within peripheral nerves.^3^ This reinforces the well‐characterized bi‐directional communication between Schwann cells and axons, especially through the Neuregulin1‐β1/ERBB2 signaling axis.^4^ Neuregulin1‐β1 (NRGβ1) is a biologically versatile protein that effects a critical role in regulating myelin thickness during peripheral nervous system (PNS) development, as well as in promoting nerve repair—particularly in the re‐myelination of axons following nerve injury.^5^, ^6^, ^7^, ^8^ This effect is mediated by either an axon‐bound form (Neuregulin1‐β1 type III),^9^ or as soluble protein via proteolytic liberation of its EGF‐like domain.^10^, ^11^ In animal models, soluble NRGβ1 has been found to rescue hypomyelination^10^ and mitigate symptoms of the hereditary neuropathy Charcot–Marie‐Tooth 1A.^12^ Importantly, clinical trials on heart failure in humans have demonstrated the safety of recombinant human NRGβ1 for clinical applications.^13^ Building on this evidence, we postulate that therapeutic administration of NRGβ1 may induce differentiation and growth arrest in undifferentiated and proliferating Schwann cells, potentially inhibiting schwannoma development.

## Materials and Methods

### Experimental Animals

All mice were handled as per local governmental and institutional animal care regulations, honoring the protocol approved by the Thueringer Landesamt fuer Verbraucherschutz, Germany (permit number 03‐011/15) or the Institutional Animal Care and Use Committee protocol 10,940. Animals had free access to food and water and were housed under constant temperature and humidity conditions on a 12/12 hour light/dark cycle. Nf2flox animals (RIKEN BioResource Centre, Japan) were used to obtain combined conditional knockout (KO) of Merlin in Schwann cells (P0‐Cre line, The Jackson Laboratory, USA, stock 017928) and neurons (Nefh‐Cre, The Jackson Laboratory, USA, stock 009102). Homozygous KO animals (P0‐Cre;Nefh‐Cre;Nf2^fl/fl^) were compared to wildtype (WT) littermates and Cre recombinase‐specific genotyping performed using the following primers: 5′‐CCA CCA CCT CTC CAT TGC AC‐3′ (forward) and 5′‐ATG TTT AGC TGG CCC AAA TG‐3′ (reverse) for P0‐Cre,^14^ as well as 5′‐GGG CCA CCG CGG ATA TAA AA‐3′ (forward) and 5′‐TGC GAA CCT CAT CAC TCG TT‐3′ (reverse) for Nefh‐Cre recombinase.^15^ All Nf2flox;P0‐Cre;Nefh‐Cre animals were on a mixed C57BL/6‐FVB/N background.

Nf2flox;Postn‐Cre mice were obtained by crossing Nf2flox animals with Postn‐Cre transgene mice. The Postn‐Cre transgene was detected by polymerase chain reaction analysis with the following primers: P1 (CAT‐TTG‐GGC‐CAG‐CTA‐AAC‐AT) and P2 (CCC‐GGC‐AAA‐ACA‐GGT‐AGT‐TA). Nf2flox;Postn‐Cre mice were on mixed FVB/NTac background.^16^

### Sciatic Nerve Crush Injury

Unilateral sciatic nerve crush injuries of Nf2flox;P0‐Cre;Nefh‐Cre mice were accomplished according to a previously described method.^17^ In brief, 8‐ to 10‐week‐old mice were anesthetized using 2% isoflurane in 100% oxygen and fur removed from 1 hind limb. After appropriate incision of the skin, the gluteal musculature was separated to reveal the right sciatic nerve. Using hemostatic forceps (Ultra Fine Haemostat; 13021‐12; tip width 0.6mm; Fine Science Tools; Germany), the nerve was crushed once through application of defined pressure for 20 seconds. The locking mechanism of the hemostatic forceps with a series of interlocking teeth ensured reproducibility and standardization of crush injury. Finally, both the gluteal musculature and skin incision were sutured using appropriate surgical suture material.

### rhNRGβ1 Treatment

The recombinant human Neuregulin1‐β1 (rhNRGβ1) purchased from Reprokine (RKQ02297; USA) is an EGF domain containing disulfide‐linked monomeric protein, consisting of 61 amino acid residues. The protein was first resolved in ddH2O, before further dilution in phosphate‐buffered saline (PBS) to reach target concentrations. For systemic administration, mice were injected intraperitoneally (ip) every other day with 10 or 20μg rhNRGβ1 per kg body weight. For focal rhNRGβ1 administration, Spongostan collagen sponges (2484887; Ethicon; Germany) were cut into cubes of 2mm edge length and incubated with 0.2mg/ml rhNRGβ1 solution for 1 hour at room temperature, before implantation. Saline solution without rhNRGβ1 served as vehicle control.

### Absorbable Collagen Sponge Performance In Vitro

Spongostan collagen sponges (2484887; Ethicon; Germany) were cut into cubes of 2mm edge length and incubated with a solution containing 0.1, 0.5, or 1.0mg/ml rhNRGβ1 for 1 hour. rhNRGβ1‐soaked sponges were transferred every day to a new dish of a 24‐well plate filled with 0.5ml PBS solution. rhNRGβ1 concentration of each dish was assessed using standard bicinchoninic acid protein assay. Sponges initially incubated with PBS only, served as control.

### Single‐Frame Motion Analysis

To evaluate locomotor function, mice were accustomed to beam‐walking in 3 to 4 trials, 1 week before surgery. This test sees the animal walk voluntarily from one end of a horizontal beam (length 1,000mm, width 40mm) toward its home cage, located at the other end of the beam. In all cases, a rear view of one walking trial was captured using a video camera—once prior surgery, then at different time‐points post‐surgery and stored in Audio Video Interleaved (AVI) format. These video sequences were examined using VirtualDub 1.6.19 software. Selected frames, in which the animals were observed in defined phases of the step cycle, were used to measure the foot base angle (FBA) as described in.^18^

### Neuropathological Assessment and Evaluation

For histological workup, paraformaldehyde‐fixed nerve samples were embedded in paraffin, cut at the site of the largest diameter (~1.5mm distal from the crush site), and mounted as a tissue micro array. A total of 4μm thick cross sections were used for hematoxylin and eosin (H&E) staining and immunohistochemical labeling of S100beta (S100b) (1:8000; Dako; GA50461‐2), myelin protein zero (MPZ) (1:300; Bioss Antibodies; bs‐0337R), p75 (1:200; Millipore; AB1554), and ErbB2 (1:500; Cell Signaling; 2,165) in an automated Ventana stainer (Ventana Medical Systems, USA), using standard antigen retrieval protocols (CC1st, no pretreatment for S100b‐protein).

### Immunoblotting

Immunoblotting was completed as described in Morrison et al,^19^ using the following primary antibodies: c‐Jun (1:1,000; Cell Signaling; 8,752), anti‐ErbB2 (1:500; Cell Signaling; 2,165), anti‐GAPDH (1:1,000; Santa Cruz; 6C5), myelin basic protein (MBP) (1:500; Millipore; MAB384), and anti‐merlin (1:500; Santa Cruz; A19).

### Dorsal Root Ganglion Tumor Volume Quantification

After fixation and decalcification in 5% formic acid, the spinal ganglia were dissected under a microscope. Tumor volume was calculated using length and width values for a particular tumor in the formula volume = length × width^2^ × 0.52, the approximate volume of a spheroid.^16^

### Quantification of Tumorlets in Nf2flox;Postn‐Cre Mice

Paraformaldehyde‐fixed (PFA) tissue blocks derived from rhNRGβ1 and control treated animals containing lower spinal cord, lumbar dorsal root ganglion (DRG), as well as distal nerve proportions, were cut in 4 planes per animal. Histological slides of H&E‐stained sections were subsequently scanned and analyzed using NanoZoomer Digital Pathology Software (Hamamatsu Photonics, Herrsching, Germany). The total area occupied by peripheral nerve tissue and DRG tissue was quantified separately in mm^2^. Likewise, neoplastic tissue regions were identified and quantified separately for peripheral nerves and DRG areas. All quantitative analyses of tumorlets in Nf2flox;Postn‐Cre mice were performed in blinded manner by 2 independent researchers (A.S. and C.H.).

### Statistical Analysis

Comparisons between groups were made in GraphPad Prism. The statistical test used is stated in the figure legends. Differences were considered significant when *p* < 0.05. All values presented as means and their standard errors.

### Additional Notes

Treatments with Nrg1 or vehicle were not administered in a blinded manner. However, the subsequent analyses were conducted in a blinded fashion. Histological quantitative analysis was performed by 2 independent researchers in a double‐blinded manner, and single‐frame motion analysis (SFMA) analysis was also carried out independently by 2 blinded researchers.

#### Randomization

Mice were randomly assigned to the experimental groups.

#### Sample Size Estimation

Because this was a pilot study, sample size calculations were not conducted in advance.

#### Data Exclusions

No outliers were excluded. One measurement was excluded because of a technical issue, where a mouse did not exhibit any movement during the SFMA analysis, preventing data collection for that subject.

## Results

### Systemic rhNRGβ1 Improves Functional Nerve Regeneration and Decreases Schwannoma Growth in Nf2flox;P0‐Cre;Nefh‐Cre Animals

**FIGURE 1:**
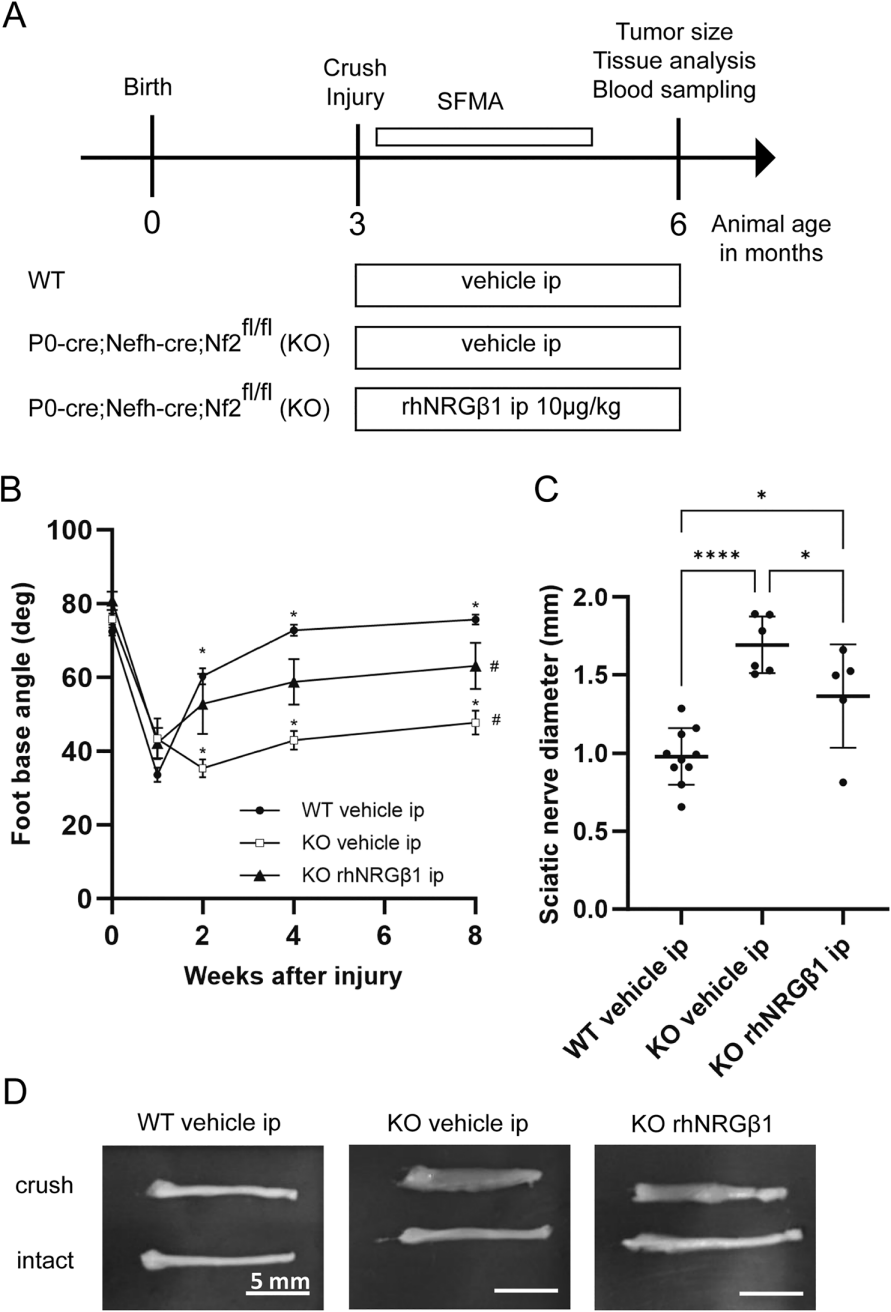
Recombinant human Neuregulin1 beta 1 (rhNRGβ1) improves functional nerve regeneration and decreases schwannoma growth. (A) Study protocol assessing the effects of systemic rhNRGβ1 on schwannoma growth following unilateral sciatic nerve crush in Nf2flox;P0‐Cre;Nefh‐Cre mice. (B) Foot base angle (FBA) quantification in wildtype (WT) mice treated with rhNRGβ1 (WT rhNRGβ1) and Nf2flox;P0‐Cre;Nefh‐Cre mutant mice receiving saline (KO vehicle) or rhNRGβ1 (knockout [KO] rhNRGβ1). FBA baseline levels were measured before injury (week 0) and functional recovery was assessed over 8 weeks (*p < 0.05 between WT and KO vehicle; #*p* < 0.05 between KO vehicle and rhNRGβ1; n = 5–10 animals per genotype, mixed‐effects model [REML] for repeated measures with Tukey's multiple comparisons test; mean ± standard error of the mean). (C) Maximum sciatic nerve diameters were quantified 3 months post‐injury in the same animals. (*p*‐value *<0.05, **<0.01, ***<0.001, ****<0.0001). (D) Images of dissected sciatic nerves from 6‐month‐old mice show crushed nerves (top) and intact nerves (bottom). Proximal parts are on the right; distal parts on the left. SFMA = single‐frame motion analysis.

Initially, we explored a therapeutic effect of NRGβ1 in treating schwannomas using the genetically engineered mouse model Nf2flox;P0‐Cre;Nefh‐Cre, where the *Nf2* gene is conditionally deleted in Schwann cells and neurons of the PNS. Conditional Nf2 KO animals (P0‐Cre;Nefh‐Cre;Nf2^fl/+^) exhibit robust schwannoma growth following single nerve crush injury with high spatiotemporal control.^20^ After performing a crush injury at the right sciatic nerve of 3‐month‐old mice, systemic administration of rhNRGβ1 (10μg per kg body weight every other day) via intraperitoneal injections was initiated (Fig 1A). Vehicle saline solution was given to KO littermates as control.

Having previously identified an extensive nerve regeneration defect in heterozygous Nf2flox^(fl/+)^;P0‐Cre;Nefh‐Cre mice after nerve injury,^20^ we confirmed our findings in homozygous mice and probed whether rhNRGβ1 administration can improve functional nerve recovery in homozygous animals (P0‐Cre;Nefh‐Cre;Nf2^fl/fl^, briefly “KO”). SFMA—a test sensitively measuring the ability of foot extension^21^—revealed that rhNRGβ1 administration significantly improves nerve recovery of KO mice compared to KO animals on saline control injections (see Fig 1B). Of note, rhNRGβ1 treatment of WT animals could not increase functional nerve regeneration compared to control injections (Fig S1).

We subsequently sought to establish whether improved nerve regeneration in rhNRGβ1‐treated KO mice is also reflected by reduced schwannoma growth. Although WT animals showed anatomically regular sciatic nerves of approximately 1mm diameter 3 months after nerve crush (see Fig 1C,D), the sciatic nerves of KO mice receiving control injections exhibited substantial enlargement of approximately 1.7‐fold gain in nerve diameter. Systemic administration of rhNRGβ1 could significantly alleviate the observed nerve enlargement in KO mice (see Fig 1C,D).

### rhNRGβ1 Administration Improves Morphological Nerve Regeneration and Induces Schwann Cell Differentiation in Nf2flox;P0‐Cre;Nefh‐Cre Animals

**FIGURE 2:**
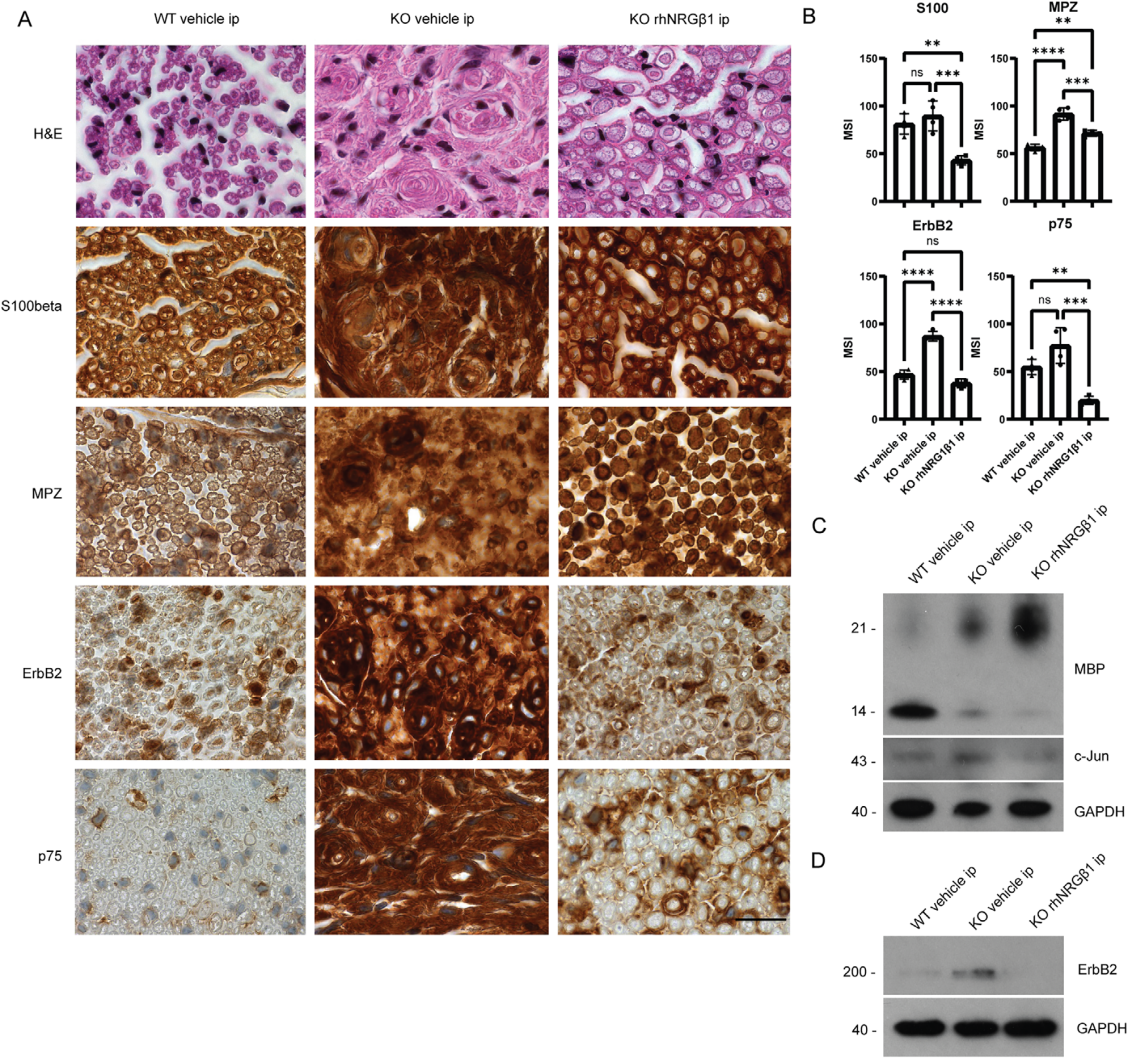
Neuropathological assessment of sciatic nerves from recombinant human Neuregulin1 beta 1 (rhNRGβ1)‐treated animals indicate Schwann cell differentiation. (A) Sciatic nerve cross sections of indicated genotypes and treatment groups 3 months after crush injury were either hematoxylin and eosin (H&E)‐stained or immunolabeled (brown color) for Schwann cell markers S100b, myelin protein zero (MPZ), the receptor tyrosine kinase ErbB2 and p75. Cell nuclei are visualized in blue. Scale bar represents 20μm. n = 2 per cohort (B) mean signal intensity (MSI) of n = 4 whole nerve sections per treatment (*p*‐value *<0.05, **<0.01, ***<0.001, ****<0.0001). (C,D) Immunoblot of sciatic nerve lysates. Pooled tissue from at least 5 different animals per indicated cohort was prepared from sciatic nerves 3 months after crush injury. (C) Immunoblot for myelin basic protein (MBP), c‐Jun, and GAPDH as loading control. (D) Immunoblot for receptor tyrosine kinase ErbB2 and GAPDH as loading control. [Color figure can be viewed at www.annalsofneurology.org]

Neuropathological assessment of nerve tissue 3 months after crush injury revealed distinct findings in different animal groups. A healthy nerve anatomy was observed in WT animals treated with control injections (Fig 2A, left panel), characterized by the regular expression of Schwann cell differentiation markers S100b and MPZ. Immunoreactivity of ErbB2 was localized to Schwann cells, indicating their involvement in the regenerative process. Scattered expression of the p75 neurotrophin receptor, a marker associated with immature (dedifferentiated) non‐myelinating Schwann cells, was also observed.

In contrast, nerves from vehicle‐treated KO animals exhibited abnormal characteristics (see Fig 2A, middle panel)—namely large clusters of disordered Schwann cells forming concentric multilayered onion bulbs. Additionally, S100b and MPZ expression levels appeared diffusely increased compared to WT mice, suggesting an aberrant Schwann cell response. The pronounced increase in ErbB2 immunoreactivity observed in these nerves may indicate aberrant signaling activity, however, additional studies are necessary to confirm this finding. Upregulation of p75 expression posits the presence of a higher number of immature (dedifferentiated) non‐myelinating Schwann cells in the vehicle‐treated KO animals.

It was notable that sciatic nerves of rhNRGβ1‐treated KO animals showed substantial structural improvement 3 months post crush injury (see Fig 2A, right panel), though some abnormalities persisted. S100b and MPZ immunoreactivity was specifically localized to Schwann cells with a myelinating phenotype, indicating partial restoration of differentiation and function. Compared with vehicle‐treated KO animals, ErbB2 expression was significantly reduced following rhNRGβ1 treatment. The treatment led to a significant reduction in the number of dedifferentiated non‐myelinating Schwann cells, as evidenced by decreased p75 immunoreactivity (see Fig 2B). However, the myelinated axon profiles in rhNRGβ1‐treated KOs remained enlarged relative to those in WT nerves, and occasional onion bulb–like structures were still apparent, albeit to a lesser extent than in vehicle‐treated KOs.

Western blot analysis of sciatic nerve tissue confirmed decisive differences between KO animals treated with either rhNRGβ1 or control injections. Although the 17 kDa MBP isoform as marker for differentiated Schwann cells is reduced in both KO groups compared to WT littermates (see Fig 2C), a 21.5‐kDa re‐myelination‐specific MBP isoform^22^, ^23^ was substantially upregulated in animals receiving rhNRGβ1. Further, the expression of c‐Jun, a transcription factor known to inhibit re‐myelination in vivo,^24^ was reduced in KO animals receiving rhNRGβ1 therapy compared to the vehicle‐treated KO cohort (see Fig 2C), however, because of limitations in blot quality, this finding should be interpreted with caution. ErbB2 shows highest expression in the latter group, but shows WT level expression following rhNRGβ1 treatment in KO mice (see Fig 2D). These results suggest that rhNRGβ1 reverses morphological nerve alterations by increasing Schwann cell differentiation in KO mice.

### Local rhNRGβ1 Application by Absorbable Collagen Sponges Reduces Schwannoma Growth

**FIGURE 3:**
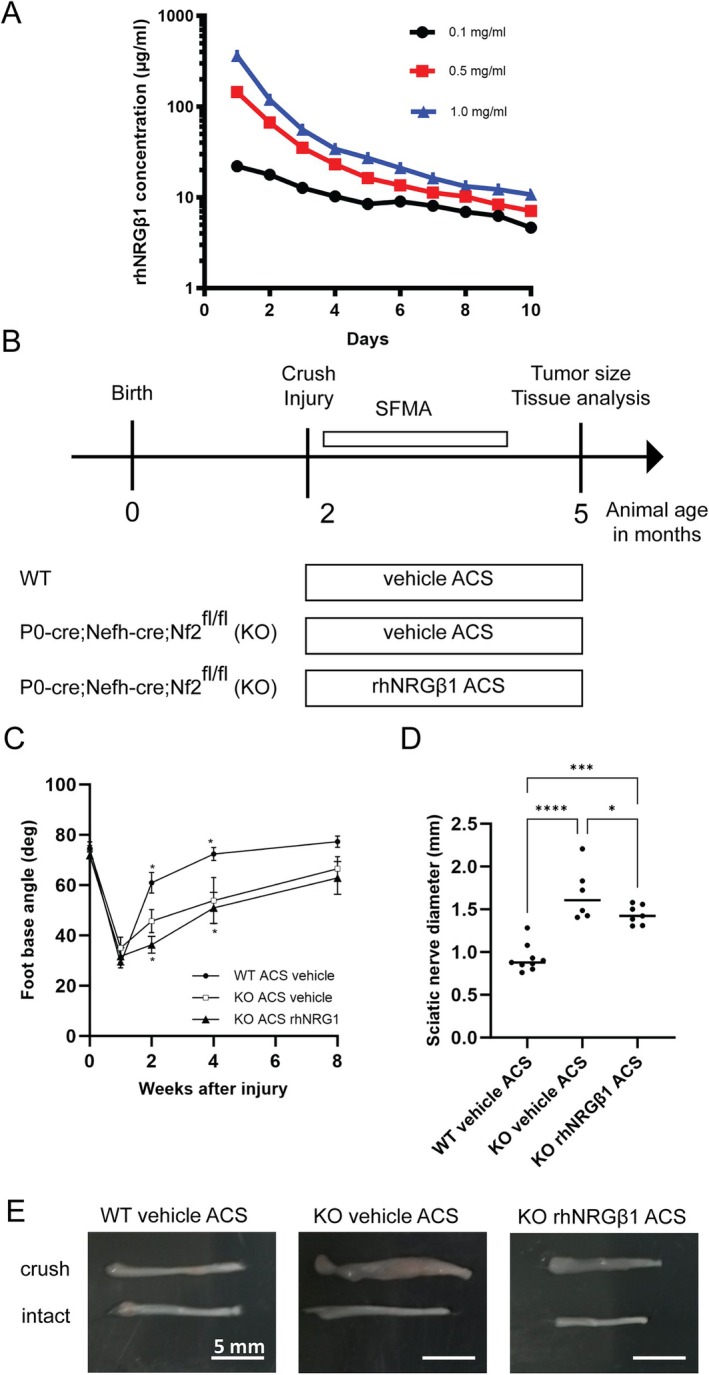
Local recombinant human Neuregulin1 beta 1 (rhNRGβ1) application via absorbable collagen sponges (ACS) reduces schwannoma growth. (A) In vitro release of rhNRGβ1 from loaded sponges was measured over time by protein concentration in phosphate‐buffered saline (PBS) after 24 hours. (B) Study protocol assessed the efficacy of local rhNRGβ1 treatment on schwannoma growth post sciatic nerve crush in Nf2flox;P0‐Cre;Nefh‐Cre mice, using ACS soaked in saline (vehicle) or rhNRGβ1. (C) Foot base angle (FBA) quantification showed functional recovery in wildtype mice with saline‐loaded sponges versus Nf2flox;P0‐Cre;Nefh‐Cre mutants treated with saline or rhNRGβ1 sponges, measured over 8 weeks (*p < 0.05; 2‐way analysis of variance for repeated measures with Sidak's post hoc test; n = 6–9 per genotype; mean ± standard error of the mean). (D) Maximum sciatic nerve diameters were quantified 3 months post‐injury in the same animals. (E) Images of dissected sciatic nerves from 5‐month‐old mice show crushed (top) and intact (bottom) nerves, with proximal parts on the right. [Color figure can be viewed at www.annalsofneurology.org]

Given that schwannomas may appear as tumors at confined locations along peripheral nerves, we examined the effectiveness of locally applied rhNRGβ1 as opposed to systemic administration. To this end, we used biodegradable and biocompatible absorbable collagen sponges (ACS) commonly used for therapeutic protein delivery^25^ and clinical hemostasis.^25^ Having evaluated the biostability of various commercially available ACS products in liquid environments at body temperature in vitro, Spongostan sponges were subsequently loaded with different concentrations of rhNRGβ1. Quantification of rhNRGβ1 released by the preloaded ACS revealed sustained protein release over several days (Fig 3A). In vivo biostability analysis in mice confirmed that the ACS remained detectable for at least 10 days after implantation adjacent to the sciatic nerves (not shown).

To assess the efficacy of locally administered rhNRGβ1, either saline‐ or rhNRGβ1‐soaked ACS cubes (2mm edge length) were positioned in close proximity to the site of the sciatic nerve crush injury in mice (see Fig 3B). In contrast to our findings on systemic rhNRGβ1 treatment, local administration via ACS could not improve functional nerve regeneration following the crush injury, as demonstrated by SFMA (see Fig 3C). However, the localized application of rhNRGβ1 using biodegradable ACS significantly reduced schwannoma growth compared to vehicle‐soaked sponges in KO mice (see Fig 3D,E).

These results indicate that the transient localized delivery of rhNRGβ1 through biodegradable ACS—most prominent in the first 48 hours—may have been sufficient to suppress early tumor‐promoting signals in Schwann cells, leading to reduced schwannoma growth. However, transient localized treatment does not improve motor functions as effectively as continuous systemic rhNRGβ1 treatment. However, the absence of sustained factor availability likely limited the support for long‐term axon–glia interactions and Schwann cell differentiation, which may explain the lack of improvement in functional nerve regeneration. Nevertheless, both treatments effectively suppress schwannoma growth. This highlights the importance of both timing and duration of rhNRGβ1 signaling for therapeutic efficacy and emphasizes the potential of this approach for targeted therapeutic interventions in schwannoma‐associated pathologies.

### rhNRGβ1 Administration Reduces Tumorlet Load in Nf2flox;Postn‐Cre Mice

A second schwannoma mouse model was deployed to further validate our finding that rhNRGβ1 reduces schwannoma growth in vivo. In Nf2flox;Postn‐Cre animals, conditional *Nf2* gene deletion is facilitated by the periostin‐Cre line, which affects gene expression in Schwann cells and neurons of the PNS.^26^ A feature of this murine NF2 disease model is that all animals spontaneously develop significant enlargement of their DRG. Moreover, microscopic schwannoma precursors, referred to as tumorlets, appear along spinal and peripheral nerves of Nf2flox;Postn‐Cre animals.^16^

**FIGURE 4:**
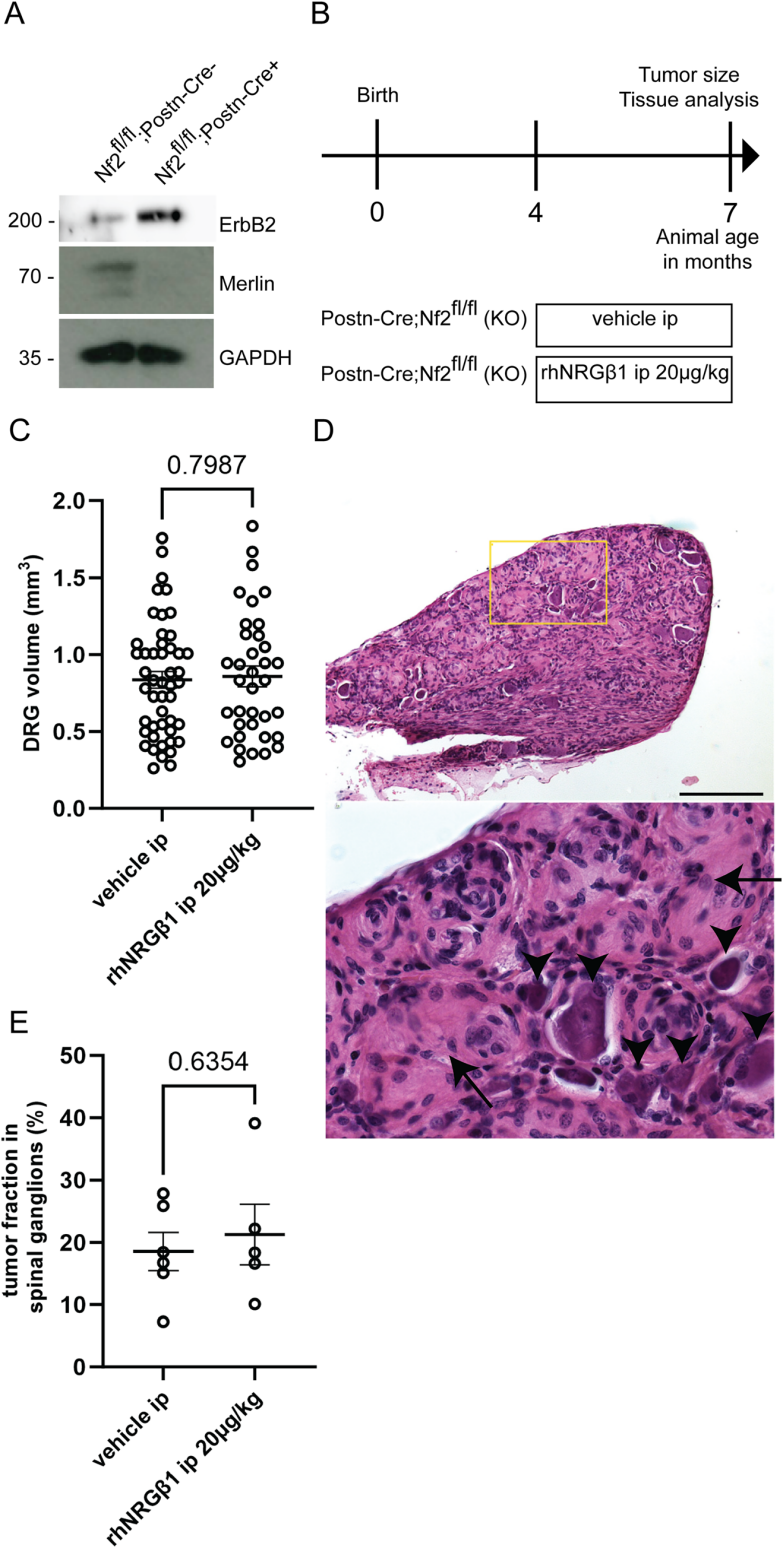
Systemic recombinant human Neuregulin1 beta 1 (rhNRGβ1) administration has no effect on dorsal root ganglion (DRG) schwannomas in Nf2flox;Postn‐Cre mutant mice. (A) Immunoblot of sciatic nerve lysates (pooled tissue from 5 different 8‐month‐old animals per indicated genotype). Immunostaining for ErbB2, Merlin (tumor suppressor protein encoded by the Nf2 gene), and GAPDH as loading control. (B) Study protocol assessing the efficacy of systemic rhNRGβ1 treatment on schwannoma growth in Nf2flox;Postn‐Cre animals. (C) DRG volume quantification of Nf2flox;Postn‐Cre mice after 3 months of rhNRGβ1 treatment. Nf2flox;Postn‐Cre mutant mice receiving intraperitoneal saline injections (vehicle ip; n = 12) were compared to mutant mice on rhNRGβ1 treatment (rhNRGβ1 ip; n = 9; mean ± standard error of the mean [SEM]; 4 DRGs analyzed per animal, 2‐tailed *t* test). (D) Representative image of a cross section (hematoxylin and eosin [H&E]‐stained) through a spinal ganglion of Nf2flox;Postn‐Cre animals displaying tumorous lesions (*arrows*) as well as DRG neurons (*arrow heads*). The lower image depicts a magnification of the selected area in the upper picture. Scale bars represent 50μm. (E) Fraction of tumor tissue within total tissue was quantified as area covered by tumor in relation to the total DRG area in investigated nerve sections. Nf2flox;Postn‐Cre mutant mice receiving intraperitoneal saline injections (vehicle ip; n = 6) were compared to mutants on rhNRGβ1 injections (rhNRGβ1; n = 5; mean ± SEM; 2‐tailed *t* test). [Color figure can be viewed at www.annalsofneurology.org]

Periostin‐Cre driven *Nf2* gene KO resulted in increased ErbB2 levels in comparison to WT littermates (Fig 4A). Four‐month‐old mutant Nf2flox;Postn‐Cre animals were treated with control or rhNRGβ1 injections intraperitoneally for 3 months (see Fig 4B). Given that a 10μg/kg dosage was well tolerated in Nf2flox;P0‐Cre;Nefh‐Cre animals, we decided to treat Nf2flox;Postn‐Cre mice at 20μg rhNRGβ1 per kg of body weight. This did not reduce the mean DRG volume compared to vehicle‐injected animals (Fig 4C). Microscopic quantification of the tissue area covered by tumorous lesions in relation to the entire DRG area (see Fig 4D), revealed the same tumor fraction in spinal ganglion of rhNRGβ1‐treated and vehicle‐injected animals (see Fig 4E). Overall, there was no effect of rhNRGβ1 treatment on DRG schwannoma development in Nf2flox;Postn‐Cre animals.

This unanticipated outcome led us to hypothesize that Schwann cells residing in DRGs might show fundamentally different responsiveness toward myelination‐inducing factors, compared to other Schwann cells found in spinal and peripheral nerves. DRG predominantly contain cell bodies of pseudounipolar sensory neurons, the vast majority of which lack myelination,^27^ whereas most peripheral nerves show a high abundance of myelinated fibers.

**FIGURE 5:**
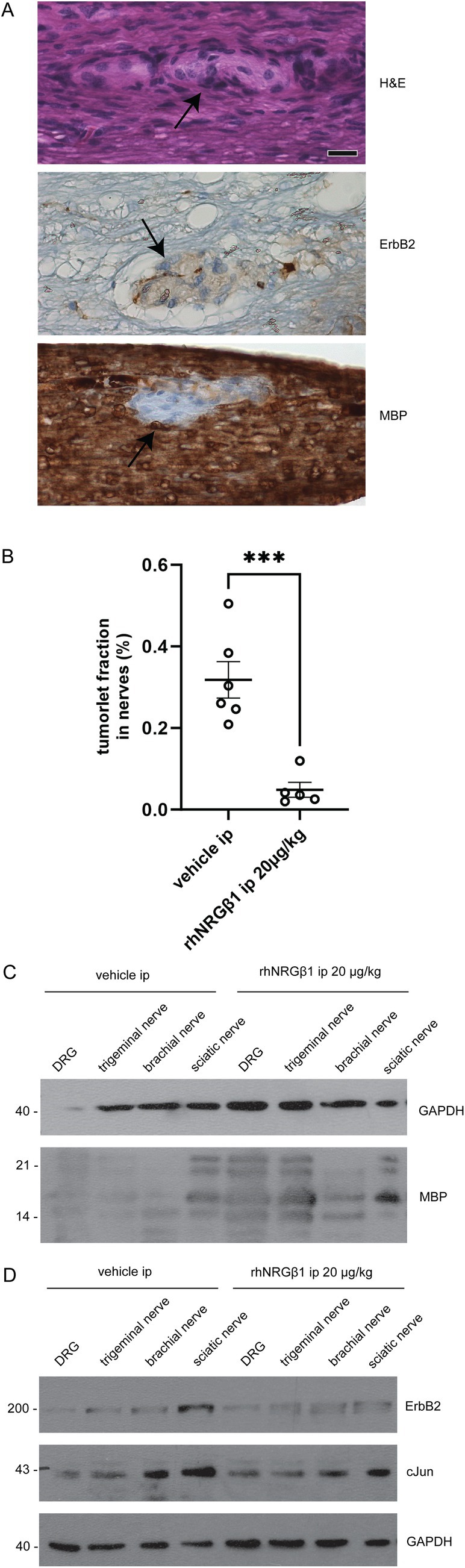
Systemic recombinant human Neuregulin1 beta 1 (rhNRGβ1) administration reduces tumorlet burden in nerves of Nf2flox;Postn‐Cre mutant mice. (A) Tumorlet‐containing nerve sections of Nf2flox;Postn‐Cre mutant animals were either hematoxylin and eosin (H&E)‐stained or immunolabeled (brown color) for the receptor tyrosine kinase ErbB2 and myelin basic protein (MBP). Cell nuclei are visualized in blue. Arrow indicates area of neoplastic Schwann cell proliferation. Scale bar represents 20μm. (B) Tumorlet fraction in nerves was quantified as area covered by tumorlet area in relation to the total area in investigated spinal and peripheral nerve sections. Nf2flox;Postn‐Cre mutant mice receiving intraperitoneal saline injections (vehicle ip; n = 6) were compared to mutants on rhNRGβ1 injections (rhNRGβ1 ip; n = 5; mean ± SEM). (C,D) Immunoblot of pooled tissue from 8 different animals prepared from sciatic nerves, brachial nerves, trigeminal nerves, and dorsal root ganglion (DRG). Nf2flox;Postn‐Cre mutant animals received either vehicle control injections (knockout [KO] vehicle ip) or rhNRGβ1 treatment (KO rhNRGβ1 ip) over 3 months. Immunostaining for MBP, ErbB2, c‐Jun, and GAPDH as loading control. [Color figure can be viewed at www.annalsofneurology.org]

We, therefore, investigated the effect of rhNRGβ1 on tumorlets along spinal and peripheral nerves of Nf2flox;Postn‐Cre mice. These areas of neoplastic Schwann cell proliferation can be detected in H&E‐stained nerve sections as disorganized cell clusters (Fig 5A). Although ErbB2 expression is enhanced here, immunostaining for MBP indicates the absence of myelin‐forming Schwann cells in tumorlets. Quantification of the nerve area affected by tumorlets in relation to the entire spinal and peripheral nerve area showed that rhNRGβ1 treatment markedly decreased their appearance (Fig 5B). Subsequent biochemical analysis of nerve tissue taken from DRG as well as trigeminal, brachial, and sciatic nerves of Nf2flox;Postn‐Cre mice, indicates that rhNRGβ1 treatment increases the expression of the MBP, particularly in sciatic nerve tissue (see Fig 5C). ErbB2 and c‐Jun levels both appear reduced following rhNRGβ1 injections, with the biggest effect seen in sciatic nerve tissue (see Fig 5D, Quantification Fig S2) These findings point toward a relevant therapeutic effect on schwannoma tumorlets within peripheral and spinal nerves following rhNRGβ1 treatment, but not in DRG of Nf2flox;Postn‐Cre.

## Discussion

Pharmacological treatment options for sporadic and tumor syndrome–associated schwannomas remain limited. Current strategies primarily rely on repurposed oncologic agents such as bevacizumab, which has demonstrated short‐ to long‐term efficacy in reducing vestibular schwannoma size and preserving hearing in some NF2 patients.^28^, ^29^, ^30^, ^31^ However, these therapies largely focus on targeting tumor vasculature or growth pathways and do not account for the unique biology of Schwann cells or their interactions with axons.^32^ Moreover, the long‐term utility of bevacizumab is often constrained by systemic side effects and variable patient responses.^33^, ^34^ Our study does not aim to position rhNRGβ1 as a direct analog or competitor to bevacizumab, but rather as a complementary or alternative approach. We propose that rhNRGβ1 represents a mechanistically distinct approach—one that targets a specific pathogenic mechanism in NF2‐SWN.

For monogenic diseases like NF2‐SWN, protein replacement therapies targeting disease‐specific proteins offer a higher probability of regulatory approval compared to biologics and small molecule drugs.^35^

This approach is supported by previous evidence demonstrating the requirement of NRGβ1 for Schwann cell‐mediated re‐myelination following nerve injury^6^, ^8^ and the reduced expression of axonal NRGβ1 in the presence of NF2 gene dosage reduction in axons.^3^ It is further speculated that schwannomas may be triggered by nerve repair processes and subsequent re‐myelination defects.^20^, ^36^

Various studies have highlighted beneficial effects of therapeutic Neuregulin1‐β1 administration in treatment of motor neuron disease,^37^ peripheral nerve regeneration,^38^ and hereditary neuropathies.^12^ Significantly, the clinical safety of rhNRGβ1 has been established through phase 2 clinical trials for heart failure.^13^

Our experiments lead us to postulate that therapeutic administration of recombinant human EGF‐like domain of Neuregulin1 beta 1 (rhNRGβ1) effectively reduces schwannoma growth in 2 distinct mouse disease models. Consistent with previous reports, our findings provide evidence that rhNRGβ1 serves as an instructive signal to Schwann cells by inducing differentiation of Schwann cells that would otherwise promote tumorigenesis. We have substantiated this claim by examining 3 molecular surrogate parameters: c‐Jun, a transcription factor known to suppress re‐myelination in vivo^24^; ErbB2, a highly expressed receptor tyrosine kinase in human schwannomas^2^; and MBP, an established marker for Schwann cell differentiation. The results showed that rhNRGβ1 treatment effectively normalized elevated levels of c‐Jun and ErbB2 in tumor‐bearing mice and induced expression of MBP, indicating Schwann cell differentiation.

In the periostin‐Cre model, the lack of a measurable therapeutic effect in the DRG likely reflects a combination of biological and anatomical factors. Importantly, we do not attribute this to a barrier to drug penetration, as the DRG compartment is known to be highly accessible because of its unique anatomical and vascular features.^39^ The DRG represents a distinct microenvironment that differs markedly from peripheral nerve, characterized by a heterogeneous mix of neuronal and non‐neuronal cells and notably limited axon–glia contacts.^40^ Unlike peripheral nerves, where robust axon–Schwann cell interactions support effective NRGβ1/ErbB2 signaling and promote Schwann cell differentiation, the anatomical context of the DRG may inherently limit the responsiveness to rhNRGβ1.^7^, ^41^ Given these constraints, we interpret the limited response in the DRG as a model‐specific limitation rather than an indication of general inefficacy of rhNRGβ1. These findings underscore the importance of anatomical context when evaluating therapies that target axon–glia signaling pathways. Nevertheless, the periostin‐Cre model remains clinically relevant, because it recapitulates key features observed in NF2‐SWN patients, including polyneuropathy and the presence of tumor‐lets.^16^, ^42^ Although these small lesions may not always progress into large schwannomas, they can contribute significantly to cumulative disease burden and progressive nerve dysfunction. Our findings suggest that rhNRGβ1 may act at this early stage to restore Schwann cell differentiation and normalize axon‐glia signaling, which could ultimately preserve nerve function and limit disease progression in patients.

rhNRGβ1, a protein comprising 61 amino acid residues with a molecular weight of 7 kDa, has been shown to readily cross both the murine blood–brain barrier and the blood–spinal cord barrier when administered intravenously.^43^ This characteristic renders intravenous administration of rhNRGβ1 a feasible option for potential therapeutic use in human individuals with vestibular schwannomas. Local administration of rhNRGβ1 using ACS or similar techniques provides an additional application possibility, especially in combination with surgical tumor resection, for the prevention of tumor recurrences. In addition, the ability of rhNRGβ1 to enhance nerve regeneration highlights its potential to preserve or restore facial nerve function in NF2‐SWN patients after vestibular schwannoma surgery.^44^ In relation to the timing of treatment, our intervention was initiated post‐crush, during the stage when tumorigenesis is triggered. It remains possible that earlier administration—before crush injury (also relevant for vestibular schwannoma surgery) may elicit an even greater therapeutic benefit. Conversely, evaluating rhNRGβ1 in the context of fully established tumors is also critical to determine its efficacy at later disease stages, which may more closely resemble clinical presentation in patients. Together, these considerations highlight important avenues for future studies to explore rhNRGβ1 in both preventive and late‐stage therapeutic settings.

In conclusion, we propose that the introduction of rhNRGβ1 as a “differentiation signal” for de‐differentiated and proliferating Schwann cells bears promise as a therapeutic approach to reducing schwannoma progression, while improving nerve function.

## Author Contributions

H.M., and A.S. contributed to the conception and design of the study; A.S., R.B., C.H., W.K.B., D.W.C., M.R., and J.B. contributed to the acquisition and analysis of data; H.M., A.S., J.B., and M.R. contributed to drafting the text or preparing the figures. [Correction added on 09 February 2026, after first online publication: Author contribution text has been revised in this version.]

## Potential Conflicts of Interest

None declared.

## Supporting information


**Figure S1 and S2.** Supporting Information.

## Data Availability

Data will be made available on request.

## References

[1] Evans DG , Howard E , Giblin C , et al. Birth incidence and prevalence of tumor‐prone syndromes: estimates from a UK family genetic register service. Am J Med Genet A 2010;152:327–332. 10.1002/ajmg.a.33139. 20082463

[2] Boin A , Couvelard A , Couderc C , et al. Proteomic screening identifies a YAP‐driven signaling network linked to tumor cell proliferation in human schwannomas. Neuro Oncol 2014;16:1196–1209. 10.1093/NEUONC/NOU020. 24558021 PMC4136892

[3] Schulz A , Kyselyova A , Baader SL , et al. Neuronal merlin influences ERBB2 receptor expression on Schwann cells through neuregulin 1 type III signalling. Brain 2014;137:420–432. 10.1093/BRAIN/AWT327. 24309211 PMC3914471

[4] Corfas G , Velardez MO , Ko CP , et al. Mechanisms and roles of axon‐Schwann cell interactions. J Neurosci 2004;24:9250–9260. 10.1523/JNEUROSCI.3649-04.2004. 15496660 PMC6730082

[5] Britsch S . The neuregulin‐I/ErbB signaling system in development and disease ‐ PubMed. Adv Anat Embryol Cell Biol 2007;190:1–65. Accessed July 18, 2022. Available at: https://pubmed.ncbi.nlm.nih.gov/17432114/. 17432114

[6] Fricker FR , Lago N , Balarajah S , et al. Axonally derived neuregulin‐1 is required for remyelination and regeneration after nerve injury in adulthood. J Neurosci 2011;31:3225–3233. 10.1523/JNEUROSCI.2568-10.2011. 21368034 PMC3059576

[7] Michailov GV , Sereda MW , Brinkmann BG , et al. Axonal Neuregulin‐1 regulates myelin sheath thickness. Science 2004;304:700–703. 10.1126/science.1095862. 15044753

[8] Stassart RM , Fledrich R , Velanac V , et al. A role for Schwann cell‐derived neuregulin‐1 in remyelination. Nat Neurosci 2013;16:48–54. 10.1038/NN.3281. 23222914

[9] Jessen KR , Mirsky R . The origin and development of glial cells in peripheral nerves. Nat Rev Neurosci 2005;2005:671–682. 10.1038/nrn1746. 16136171

[10] Fleck D , van Bebber F , Colombo A , et al. Dual cleavage of neuregulin 1 type III by BACE1 and ADAM17 liberates its EGF‐like domain and allows paracrine signaling. J Neurosci 2013;33:7856–7869. 10.1523/JNEUROSCI.3372-12.2013. 23637177 PMC6618983

[11] Syed N , Kim HA . Soluble neuregulin and Schwann cell myelination: a therapeutic potential for improving remyelination of adult axons. Mol Cell Pharmacol 2010;2:161–167. 10.4255/MCPHARMACOL.10.22. 21274416 PMC3026321

[12] Fledrich R , Stassart RM , Klink A , et al. Soluble neuregulin‐1 modulates disease pathogenesis in rodent models of Charcot‐Marie‐tooth disease 1A. Nat Med 2014;20:1055–1061. 10.1038/NM.3664. 25150498

[13] Mendes‐Ferreira P , De Keulenaer GW , Leite‐Moreira AF , Brás‐Silva C . Therapeutic potential of neuregulin‐1 in cardiovascular disease. Drug Discov Today 2013;18:836–842. 10.1016/J.DRUDIS.2013.01.010. 23384772

[14] Feltri ML , D'Antonio M , Previtali S , et al. P0‐Cre transgenic mice for inactivation of adhesion molecules in Schwann cells. Ann N Y Acad Sci 1999;883:116–123. 10.1111/j.1749-6632.1999.tb08574.x. 10586237

[15] Hirasawa M , Cho A , Sreenath T , et al. Neuron‐specific expression of Cre recombinase during the late phase of brain development. Neurosci Res 2001;40:125–132. 10.1016/S0168-0102(01)00216-4. 11377750

[16] Gehlhausen JR , Park SJ , Hickox AE , et al. A murine model of neurofibromatosis type 2 that accurately phenocopies human schwannoma formation. Hum Mol Genet 2015;24:1–8. 10.1093/HMG/DDU414. 25113746 PMC4262489

[17] Bauder AR , Ferguson TA . Reproducible mouse sciatic nerve crush and subsequent assessment of regeneration by whole mount muscle analysis. J Vis Exp 2012;60:e3606. 10.3791/3606. PMC3376939 22395197

[18] Schulz A , Büttner R , Toledo A , et al. Neuron‐specific deletion of the Nf2 tumor suppressor impairs functional nerve regeneration. PLoS One 2016;11:e0159718. 10.1371/JOURNAL.PONE.0159718. 27467574 PMC4965074

[19] Morrison H , Sherman LS , Legg J , et al. The NF2 tumor suppressor gene product, merlin, mediates contact inhibition of growth through interactions with CD44. Genes Dev 2001;15:968–980. 10.1101/gad.189601. 11316791 PMC312675

[20] Schulz A , Büttner R , Hagel C , et al. The importance of nerve microenvironment for schwannoma development. Acta Neuropathol 2016;132:289–307. 10.1007/s00401-016-1583-8. 27236462 PMC4947119

[21] Fey A , Schachner M , Irintchev A . A novel motion analysis approach reveals late recovery in C57BL/6 mice and deficits in NCAM‐deficient mice after sciatic nerve crush. J Neurotrauma 2010;27:815–828. 10.1089/NEU.2009.1217. 20121417

[22] Capello E , Voskuhl RR , McFarland HF , Raine CS . Multiple sclerosis: re‐expression of a developmental gene in chronic lesions correlates with remyelination. Ann Neurol 1997;41:797–805. 10.1002/ANA.410410616. 9189041

[23] He X , Knepper M , Ding C , et al. Promotion of spinal cord regeneration by neural stem cell‐secreted trimerized cell adhesion molecule L1. PLoS One 2012;7:e46223. 10.1371/JOURNAL.PONE.0046223. 23049984 PMC3458024

[24] Fazal SV , Gomez‐Sanchez JA , Wagstaff LJ , et al. Graded elevation of c‐Jun in Schwann cells in vivo: gene dosage determines effects on development, remyelination, tumorigenesis, and hypomyelination. J Neurosci 2017;37:12297–12313. 10.1523/JNEUROSCI.0986-17.2017. 29109239 PMC5729195

[25] Friess W . Collagen‐‐biomaterial for drug delivery. Eur J Pharm Biopharm 1998;45:113–136. 10.1016/S0939-6411(98)00017-4. 9704909

[26] Sonnenberg‐Riethmacher E , Miehe M , Riethmacher D . Promotion of periostin expression contributes to the migration of Schwann cells. J Cell Sci 2015;128:3345–3355. 10.1242/JCS.174177/260004/AM/PROMOTION-OF-PERIOSTIN-EXPRESSION-CONTRIBUTES-TO. 26187852

[27] Tandrup T . Are the neurons in the dorsal root ganglion pseudounipolar? A comparison of the number of neurons and number of myelinated and unmyelinated fibres in the dorsal root. J Comp Neurol 1995;357:341–347. 10.1002/CNE.903570302. 7673472

[28] Plotkin SR , Merker VL , Halpin C , et al. Bevacizumab for progressive vestibular schwannoma in neurofibromatosis type 2: a retrospective review of 31 patients. Otol Neurotol 2012;33:1046–1052. 22805104 10.1097/MAO.0b013e31825e73f5

[29] Alanin MC , Lassmann R , Schrøder H , et al. The effect of bevacizumab on vestibular schwannoma tumour size and hearing in patients with neurofibromatosis type 2. J Neurooncol 2015;124:147–155. 10.1007/s11060-015-1780-9. 25421643

[30] Mautner VF , Nguyen R , Kutta H , et al. Bevacizumab induces regression of vestibular schwannomas in patients with neurofibromatosis type 2. Neuro Oncol 2009;12:14–18. 20150363 10.1093/neuonc/nop010 PMC2940556

[31] Blakeley JO , Ye X , Duda DG , et al. Efficacy and biomarker study of bevacizumab for hearing loss resulting from Neurofibromatosis type 2 associated vestibular schwannomas. J Clin Oncol 2016;34:1669–1675. 26976425 10.1200/JCO.2015.64.3817 PMC4872317

[32] Baser ME et al. Anti VEGF treatment improves neurological function and augments radiation response in NF2 schwannoma model. Proc Natl Acad Sci U S A 2016;113:E7149–E7158. 10.1073/pnas.1512570112 PMC4664377 26554010

[33] Shi J , Lu D , Gu R , et al. Reliability and toxicity of bevacizumab for neurofibromatosis type 2 related vestibular schwannomas: a systematic review and meta‐analysis. Am J Otolaryngol 2021;42:103148. 34214711 10.1016/j.amjoto.2021.103148

[34] Screnci M , Puechmaille M , Berton Q , et al. Bevacizumab for vestibular schwannomas in Neurofibromatosis type 2: a systematic review of tumor control and hearing preservation. J Clin Med 2024;13:7488. 10.3390/jcm13237488. 39685944 PMC11642482

[35] Gorzelany JA , De Souza MP . Protein replacement therapies for rare diseases: a breeze for regulatory approval? Sci Transl Med 2013;5:178fs10. 10.1126/SCITRANSLMED.3005007. 23536010

[36] Mindos T , Dun X , North K , et al. Merlin controls the repair capacity of Schwann cells after injury by regulating hippo/YAP activity. J Cell Biol 2017;216:495–510. 10.1083/jcb.201606052. 28137778 PMC5294779

[37] Lasiene J , Komine O , Fujimori‐Tonou N , et al. Neuregulin 1 confers neuroprotection in SOD1‐linked amyotrophic lateral sclerosis mice via restoration of C‐boutons of spinal motor neurons. Acta Neuropathol Commun 2016;4:15. 10.1186/S40478-016-0286-7. 26891847 PMC4758105

[38] Gambarotta G , Ronchi G , Geuna S , Perroteau I . Neuregulin 1 isoforms could be an effective therapeutic candidate to promote peripheral nerve regeneration. Neural Regen Res 2014;9:1183–1185. 10.4103/1673-5374.135324. 25206780 PMC4146285

[39] Lund H , Hunt MA , Kurtović Z , et al. CD163+ macrophages monitor enhanced permeability at the blood–dorsal root ganglion barrier. J Exp Med 2024;221(2):e20230675. 38117255 10.1084/jem.20230675 PMC10733632

[40] Haberberger RV , Barry C , Dominguez N , Matusica D . Human Dorsal Root Ganglia. Front Cell Neurosci 2019;13:271. 31293388 10.3389/fncel.2019.00271 PMC6598622

[41] Jessen KR , Mirsky R . The repair Schwann cell and its function in regenerating nerves. Nat Rev Neurosci 2016;17:281–296. 10.1038/nrn.2016.21. PMC4929314 26864683

[42] Stemmer‐Rachamimov AO , Ino Y , Lim ZY , et al. Loss of the NF2 gene and merlin occur by the tumorlet stage of schwannoma development in neurofibromatosis 2. J Neuropathol Exp Neurol 1998;57:1164–1167. 9862639 10.1097/00005072-199812000-00008

[43] Kastin AJ , Akerstrom V , Pan W . Neuregulin‐1‐beta1 enters brain and spinal cord by receptor‐mediated transport. J Neurochem 2004;88:965–970. 10.1046/J.1471-4159.2003.02224.X. 14756818

[44] Rinaldi V , Casale M , Bressi F , et al. Facial nerve outcome after vestibular schwannoma surgery: our experience. J Neurol Surg B Skull Base 2012;73:21–27. 10.1055/S-0032-1304559. 23372991 PMC3424019

