# Supplementary material for: Recombinant Human Neuregulin1‐β1 Significantly Reduces Schwannoma Growth in Mice
Source: Ann Neurol. 2025 Oct 17;99(2):369–81. doi: 10.1002/ana.78050 (PMC12894486; doi:10.1002/ana.78050)
Supplement: Supplementary file 1 — Figure S1 and S2. Supporting Information. [file ANA-99-369-s001.docx]

**Supplementary Material**

Fig. S1


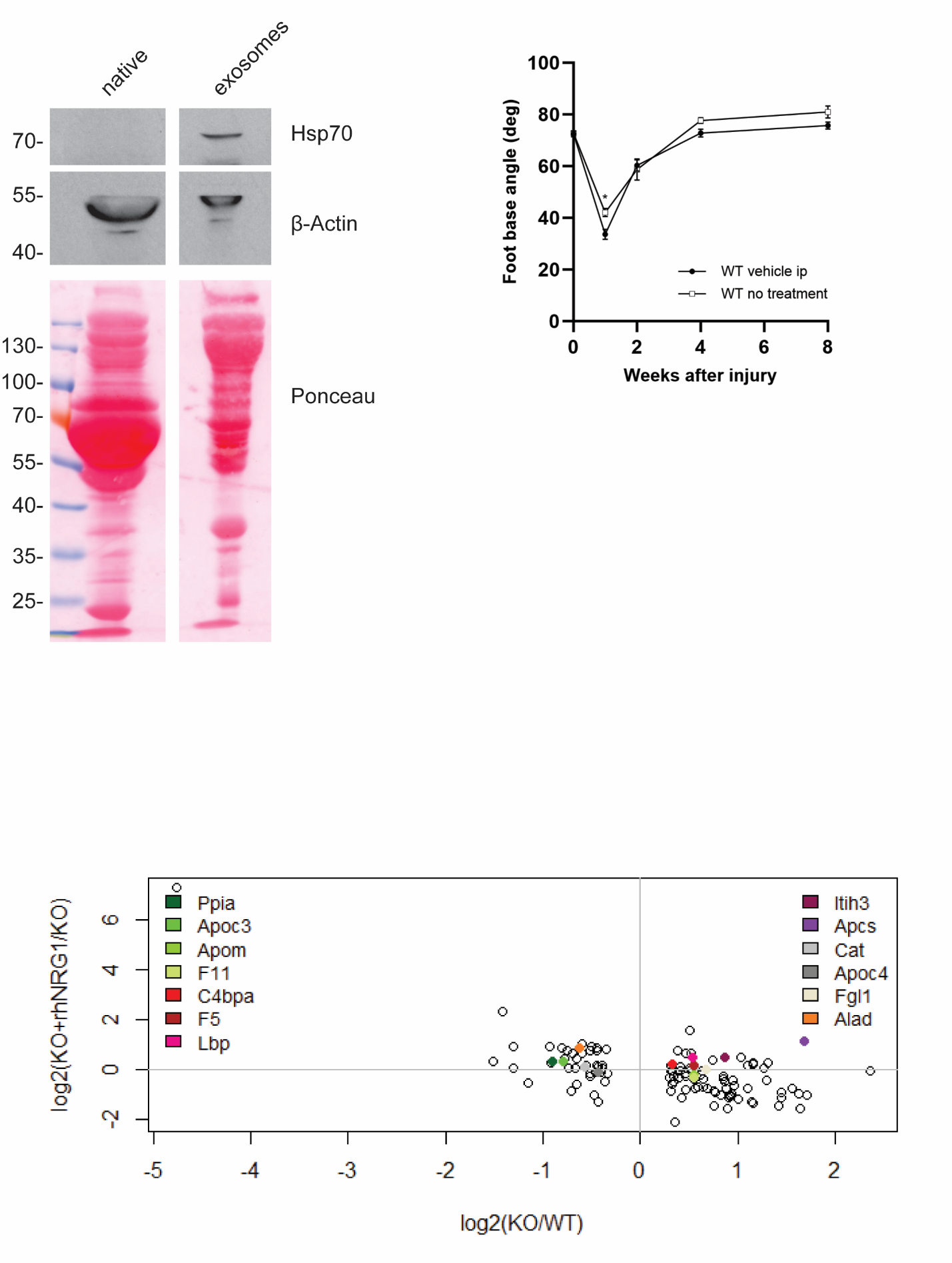


**Fig. S1.** **Foot-base angle (FBA) quantification of wildtype mice receiving intraperitoneal administration of rhNRGβ1 (WT rhNRG1), or no treatment.** FBA baseline levels were measured before nerve injury (week 0). Functional motor recovery after sciatic nerve crush was assessed for 8 consecutive weeks (* P < 0.05 for differences between WT and KO treated with the vehicle; # P < 0.05 for differences between KO treated with vehicle or rhNRGβ1; Mixed-effects model (REML) for repeated measures with Tukey´s multiple comparisons test (n = 5-10 animals per genotype; mean ± SEM).

Fig. S2


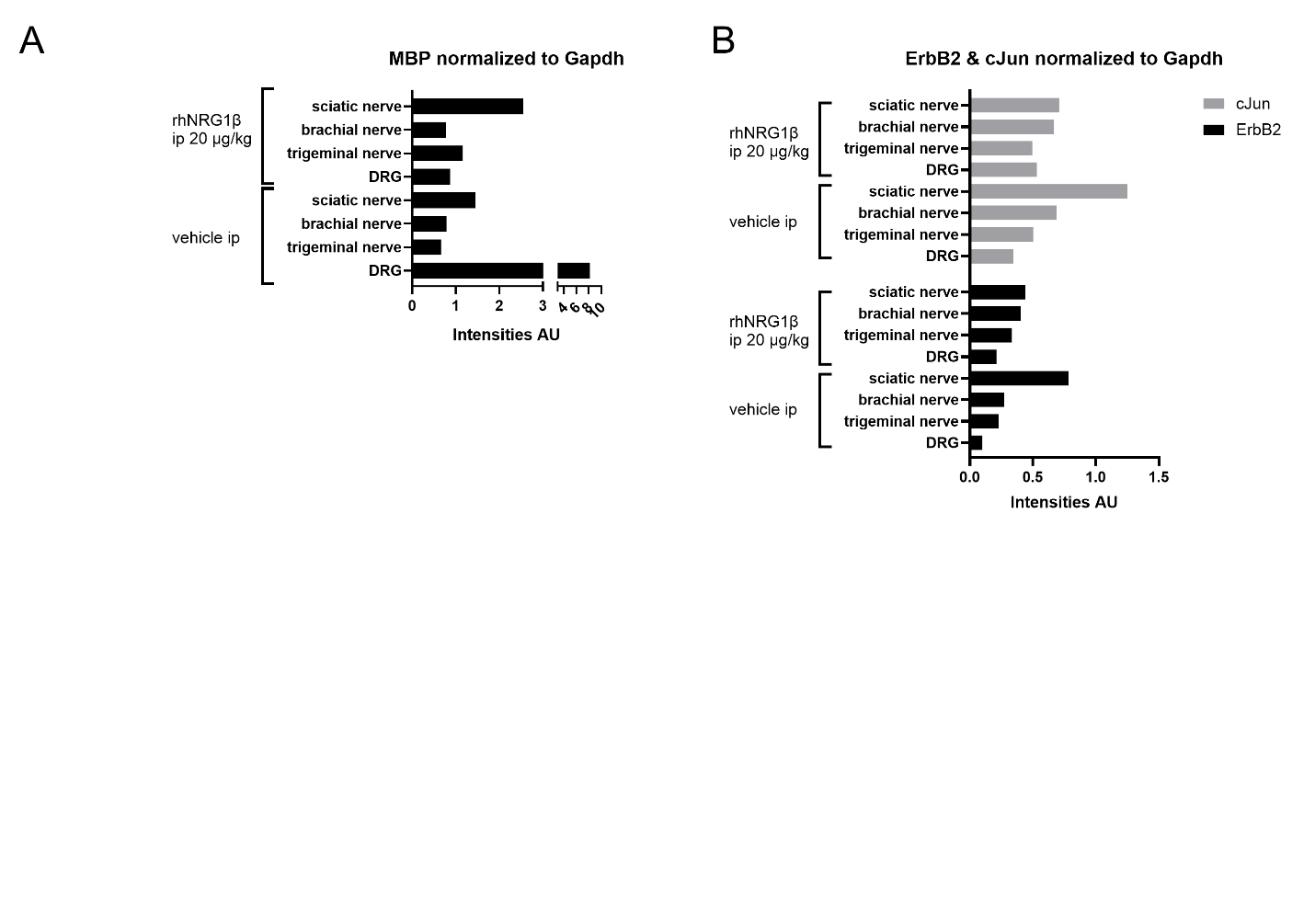


**Fig. S2. Densitometric quantification of Immunoblots blots from Fig 5 C and D. Band intensities as arbitrary units (AU)**
